# Recent advances in managing gastrointestinal stromal tumor

**DOI:** 10.12688/f1000research.11118.1

**Published:** 2017-09-14

**Authors:** Florence Duffaud, Axel Le Cesne

**Affiliations:** 1 Service d’Oncologie Médicale, CHU La Timone, Marseille, France; 2 UMR S910 INSERM, Marseille, France; 3 Aix-Marseille Université, Marseille, France; 4 Département d’Oncologie Médicale, Gustave Roussy Institut, Villejuif, France

**Keywords:** gastrointestinal stromal tumours, tyrosine kinase inhibitors, microscopic complete resection

## Abstract

Constitutive activating mutations in *KIT *and platelet-derived growth factor receptor α ( *PDGFRα*) are heavily involved in the pathobiology of gastrointestinal stromal tumors (GISTs). This disease has served as an effective “proof-of-concept” model for targeting gain-of-function kinase mutations in cancer. This review discusses the current standard of care in terms of pharmacotherapy in the management of localized and metastatic GISTs.

## Introduction

Gastrointestinal stromal tumors (GISTs) are the most common mesenchymal neoplasms that arise in the gastrointestinal tract. Surgery is the cornerstone of treatment for primary localized tumors which can easily be resected without inducing functional deficits. Prior to the advent of the first tyrosine kinase inhibitors (TKIs) like imatinib, there were few treatment options available to patients with advanced GIST, and their prognosis was dismal, with survival generally measured in weeks to a few months. This article presents an evaluation of current GIST management, discusses important practice points that may impact upon questions of therapy for primary and metastatic GISTs, emphasizes the most recent advances in the field, and discusses emerging steps to prevent and improve the outcomes for TKI-refractory disease.

## Advanced and metastatic GIST management

### Imatinib as first-line treatment for advanced/metastatic GISTs

Imatinib has been considered the standard first-line therapy for inoperable or metastatic GISTs since its approval in 2002. It is an inhibitor of a few kinases including KIT, platelet-derived growth factor receptor α (PDGFRA), ABL, and CSF1R. The standard dose is 400 mg/day. A higher dosage (800 mg/day) for *KIT* exon 9-mutated GISTs was endorsed by the NCCN and ESMO guidelines ^1, 2^, as it demonstrated a significant progression-free survival (PFS) advantage (HR 0.89, 95% CI 0.79–1.00) despite the fact that no difference in overall survival (OS) was observed ^3, 4^. For patients treated with first-line imatinib for advanced/metastatic GIST, half to two-thirds experience an objective response after imatinib treatment, according to RECIST, with a median time to response of 3–4 months. Despite the important benefit in most patients, the median time to progression (TTP) is approximately 24 to 30 months ^5– 7^, and median OS is approximately 57–60 months ^5– 7^. Nearly 50% of patients survive for more than 5 years, irrespective of imatinib starting dose, with approximately 15–23% of patients showing a durable response lasting for more than 10 years ^4– 10^. Patients with stable disease lasting for more than 6 months show favorable survival outcomes comparable to those with objective responses ^8^. However, 10–15% of metastatic GIST patients show intolerance or primary resistance to imatinib (defined as progressive disease within 3 months of imatinib initiation). These tumors most commonly are those with mutations in *PDGFRA*, particularly the D842V mutation in exon 18, or those lacking mutations in either *KIT* or *PDGFRA*.

### Does the type of mutation affect prognosis in metastatic GISTs?

Multiple trials have confirmed the superior prognosis of patients with advanced GISTs who have a *KIT* exon 11 mutation and are treated with imatinib, compared with other mutation subtypes ^6, 10, 11^. Upon analysis of the period of response in the common mutation subgroups, exon 11 mutations demonstrated the longest PFS, and exon 9 mutations or patients lacking both *KIT* and *PDGFRA*, i.e. the variant which once was called “wild-type” mutations, exhibited less favorable PFS ^4, 11, 12^. Since the majority of “wild-type” GISTs are now identified as having genetic or epigenetic loss of succinate dehydrogenase (SDH) subunits, the more accurate term is generally “SDH-deficient GIST”, unless there is some other rare mutation such as *BRAF V600E, NF1* deletions, or *NTRK* fusions. OS was also different in those with a *KIT* exon 11 mutation compared to those with exon 9 mutated or “wild-type” GIST, but no significant difference in OS has been reported between exon 9 mutated and wild-type GIST patients. Complete responses to TKI therapy are very rare.

A large proportion of patients with advanced GISTs in whom imatinib is useful demonstrate persistent measurable disease and ultimately develop progressive disease, usually within 2–3 years. The most common mechanism of resistance to TKIs in patients with GISTs occurs because of clonal evolution. These clones express the primary mutation along with additional mutations that render them resistant to imatinib, leading to treatment failure and progression of disease (most often in pre-existing sites of bulk disease); the secondary resistance mutations occur in the same gene that was originally activated by mutation. The most common secondary mutations occur in two regions of the KIT protein: the ATP-binding pocket (encoded by exons 13 and 14) and the kinase activation loop (encoded by exons 17 and 18) ^13– 15^.

### For how long should imatinib treatment be continued?

The French BFR14 trial addressed the question of imatinib dosing interruption in metastatic GISTs following initial disease controls after 1, 3, and 5 years of daily treatment with 400 mg of imatinib in non-progressive patients who have not yet developed progressive disease. In patients randomized to stop imatinib dosing, the median PFS after initial disease control of 1, 3, or 5 years was quite short (only 7, 9, or 13 months, respectively); in comparison, the patients randomized to continue imatinib dosing continued to maintain disease control with a median PFS of 29 months for the group randomized after the first year of disease control, and median PFS was much longer and not reached at the time of the report in patients randomized after 3 or 5 years of disease control ^16– 18^. Although the majority of patients who progressed after dosing interruption were able to regain control of the progressive disease, a small number of patients had continuation of progression, and this led to the standard of care to avoid lengthy dosing interruptions of imatinib in patients with disease stability or response. The worldwide standard is that imatinib should optimally be continued in metastatic GIST until disease progression, even for patients who may have obtained a complete response via radical excision of residual GIST masses during imatinib treatment.

The French BFR14 study also asked what the effect of long-term continuous imatinib therapy was on the evolution of secondary resistance ^18^. The endpoint of time to secondary resistance (TSR) was defined as the time to disease progression while on imatinib dosing. The 2-year PFS following randomization (in the continuous treatment arm) increased from 62% (in the patients randomized after 1 year of initial disease control) to 80% (for those patients randomized after 3 years of disease control ^19^). Of course, the evolution of secondary resistance is a function of the individual’s disease biology as well as the length of continuous control of imatinib. These results demonstrate that the rate of secondary resistance decreases over time, suggesting the possibility of long-term tumor control with continuous imatinib in a significant subset of patients with metastatic GISTs. These results were also reported in the most recent update from the long-term results of the B2222 phase II trial ^20^.

### What is the appropriate therapy with evidence of disease progression on imatinib?

***Prolongation of imatinib therapy by dose escalation.*** Three randomized trials performed early in the clinical development of imatinib for GIST have demonstrated a benefit with a higher dose of imatinib in patients progressing on 400 mg per day. Clinical results from the EORTC and SWOG studies ^3, 5, 21^, in which patients were allowed to crossover to higher-dose imatinib (800 mg/day) after initial progression, revealed that disease was controlled for some period of time in approximately one-third of patients after dose escalation. The median PFS following progression and crossover to a higher dose of imatinib was 3 and 5 months in the EORTC and SWOG trials, respectively ^3, 5, 21^. A meta-analysis of these combined results indicated that the benefit from such dose escalation was virtually all in the subset of patients whose GIST harbored *KIT* exon 9 mutations ^11^. Additionally, long-term data in the B2222 phase II trial demonstrated that approximately 25% of patients progressing on a low dose benefited from a dose increase ^6, 20^. Consequently, a dose increase to 400 mg twice daily is an option when imatinib resistance develops, especially for patients with a low plasma imatinib concentration ^22, 23^.

### Sunitinib: second-line treatment in patients with GIST after failure or intolerance to imatinib

Since the failure of imatinib, sunitinib malate (Sutent), a multi-target TKI with powerful activity against *KIT and PDGFRA*, as well as a number of other kinases, has demonstrated efficacy as a second-line therapy and is now approved globally for use in metastatic GIST in patients who are resistant or intolerant to imatinib based on the results of a double-blind, placebo-controlled phase III trial ^24^. In this study, 312 patients were enrolled and randomized to receive sunitinib or placebo. Sunitinib dose was 50 mg daily administered on a schedule of 4 weeks on and 2 weeks off. Despite a very low objective response rate (7%) in the sunitinib arm, the median PFS rates were 6.3 and 1.5 months in the sunitinib and placebo arms, respectively (HR 0.33, *p*<0.0001). The primary end-point, TTP, was fourfold higher in the sunitinib arm compared with placebo (27 versus 6 weeks, respectively). Despite crossover, OS was better, even though it was not significant, in the sunitinib arm than in the placebo arm (HR 0.49). The most frequent treatment-related adverse events were fatigue, diarrhea, hand-foot syndrome, and hypothyroidism ^24^.

In addition, a phase II single arm looked at the feasibility of a daily dosing of sunitinib at a continuous dose of 37.5 mg ^23^. The clinical benefit rate was 53%, with a median PFS of 34 weeks and a median OS of 107 weeks, and the toxicity profile was similar to that seen in the phase III study. The continuous use of 37.5 mg daily was later approved in the US and EU and is considered an alternative dosing schedule ^25^. Similar to the mechanisms of acquired secondary resistance with imatinib, resistance to sunitinib can be explained in large part by the development of other secondary mutations in *KIT* which allow the kinase to escape the inhibitory action of sunitinib. The assessments of tumor genotype following imatinib failure can predict the sensitivity of resistance to sunitinib, to a great extent. The degree of disease control, including length of PFS and median OS, was noted to be significantly higher in patients whose GIST was characterized by a primary exon 9 mutation in *KIT* or those with no mutations in either *KIT* or *PDGFRA.* Secondary mutations were also correlated with activity of sunitinib with sensitivity in the gatekeeper mutations in the *KIT* ATP-binding pocket, whereas mutations in the exon 17 *KIT* region encoding the kinase activation loop were generally resistant to sunitinib as well as imatinib ^26^.

### Regorafenib: a recent standard of care for metastatic GISTs

Regorafenib is an oral TKI that inhibits numerous kinases that participate in oncogenesis (KIT, PDGFRA, RET, RAF1, and BRAF V600E), angiogenesis (VEGFR1–3 and TIE2), and the tumor microenvironment (PDGFR and FGFR) ^27^. In a phase II trial that included 33 patients with GISTs resistant to imatinib and sunitinib but who were sorafenib naive, regorafenib at a dose of 160 mg daily for 3 weeks, in a 4-week cycle, showed a clinical benefit rate of 79% (95% CI 61–91%) and a median PFS of 10 months ^28^. Long-term follow-up (FU) results of this phase II trial, with a median FU of 41 months, reported a median PFS of 13.2 months (95% CI 9.2–18.3 months) and a median OS of 25 months (95% CI 13.2–39.1 months) and showed that patients whose tumors harbored a *KIT* exon 11 mutation demonstrated the longest median PFS (13.4 months), whereas patients with *KIT/PDGFRA* wild-type and non-SDH complex (non-SDH)-deficient tumors experienced a median PFS of 1.6 months ( *p*<0.0001) ^29^.

Based on these promising results, a randomized (2:1), double-blind, placebo-controlled phase III study ^30^ was performed in patients with GISTs resistant to both imatinib and sunitinib; the primary end-point was PFS. A total of 199 patients were allocated to regorafenib 160 mg/day or placebo (3 weeks on and 1 week off) until disease progression, along with best supportive care in both arms. Patients were unblinded at the time of disease progression; however, those on placebo were able to crossover to the study drug if permitted by the investigator ^30^. The results of this trial favored regorafenib, with median PFS of 4.8 months for patients initially randomized to regorafenib compared with 0.9 months for those randomized initially to the placebo arm (HR 0.27, 95% CI 0.19–0.39, *p*<0.0001). Given the rapidity of the disease progression and the fact that the majority of patients on placebo were able to crossover to regorafenib on unblinding, it is not surprising that no difference in OS was observed between the two study arms. The clinical benefit of regorafenib was studied in sensitivity testing and found to be consistent across all prospectively identified subsets of patients, except for the very small subset of patients with primary resistance to imatinib (defined as those whose initial duration of imatinib treatment was <6 months). Regorafenib-related adverse events grade III or higher were reported in 61% of patients, and the most common were hypertension and hand-foot skin reaction. A total of 58% of patients required dose interruption and 50% required dose reductions for adverse events, but there was little occurrence of treatment discontinuation ^30^. Based on these results, regorafenib was approved as a third-line standard of care in metastatic GISTs after failure or intolerance to imatinib and sunitinib.

### Imatinib rechallenge as an alternative strategy after failure of imatinib and sunitinib

Despite the remarkable advances in the therapeutic options for metastatic GISTs, the overwhelming majority of patients will develop resistance to all available TKI therapies and exhibit refractory disease progression despite all TKIs. Re-introduction of imatinib dosing has been a common practice despite prior failure, justified by evidence that rapidly symptomatic disease progression can be a nearly universal occurrence if all TKI therapy is discontinued. There is evidence that some bulky subsets of tumor cells are controlled by any TKI therapy and that the withdrawal of all TKI suppression allows the entirety of tumor clones to expand rapidly. A randomized, double-blind, placebo-controlled, phase III trial known as RIGHT was performed in Korea to formally assess the potential benefits of imatinib resumption in patients with imatinib-refractory GISTs following progression after at least imatinib and sunitinib, with the caveat that they must demonstrate prior initial benefit from imatinib in the first-line setting ^31^. Following disease progression, a total of 81 patients were randomized to receive either imatinib 400 mg daily or matching placebo. Imatinib was well tolerated, and median PFS with imatinib was twice that for patients receiving placebo (1.8 versus 0.9 months, respectively), together with a disease control rate at 12 weeks of 32%. There was no notable improvement in OS. Thus, the benefit seen with the rechallenge of imatinib in TKI-refractory GIST has been suggested to be caused by the constant kinase inhibition of the majority of disease clones that have no acquired mutations; however, the short PFS indicates that resistant clones still grow. The advantages of imatinib rechallenge offset its toxicities, reinforcing its clinical significance for patients without active treatment options available to minimize symptom-worsening GISTs, especially since quality of life was not impaired by imatinib in this fragile population ^32^.

## Role of surgery in metastatic GIST

Most patients reach partial response or stable disease on imatinib, but around half acquire secondary resistance after 2 years. Existing data indicate that cytoreductive surgery could be a possibility in imatinib-responsive patients with metastatic GISTs, especially if there is successful complete (R0/R1) resection of residual metastatic disease ^1, 33– 37^. The value of surgery in patients with focal tumor progression being treated with imatinib is uncertain, but this approach is a possible option. Recently, the role of cytoreductive surgery for metastatic GISTs treated with TKIs was reported in a two-institution large analysis after 400 operations were performed on 323 patients ^35^. The authors concluded that surgery in metastatic GIST patients in the absence of multifocal progressive disease on imatinib is associated with outcomes that are at least comparable with second-line sunitinib and may be considered in selected patients ^35^.

Overall, patients with multifocal progression undergoing surgery have a poor result. Surgery does not seem to benefit patients with generalized disease progression on imatinib ^37^ and should not be offered unless as an emergency where palliative intervention may be justified ^35^. Although surgery is a viable option for metastatic GIST patients treated with sunitinib, it is common for incomplete resections to occur, there is a high chance of complications, and there is uncertainty regarding the survival benefit ^33^. Clinicians must adopt a considered multidisciplinary consultation to establish the best local treatment choices in metastatic GIST patients, on an individual basis, after sharing the decision with the patient ^33^.

### Localized GISTs

Microscopic complete resection with histologically negative margins (R0) without rupturing the tumor is the standard treatment for localized GISTs ^1, 2, 38^. Although a significant proportion of patients will be cured with surgery alone, approximately 40% will eventually relapse, the great majority within the first 5 years ^38– 40^. These outcomes underscore the need for adjuvant therapy. Given the efficacy of imatinib in the metastatic setting, the use of imatinib has been extended to the adjuvant setting for the treatment of adult patients following GIST resection ^41^.

## Risk factors associated with GIST recurrence

Independent prognostic factors for GIST include tumor size and site, mitotic count, and tumor rupture ^40^. Risk stratification is essential to identify and better define the patients with GIST who are most likely to benefit from adjuvant imatinib therapy ^42, 43^. Among the risk-stratification schemes currently available for operable GISTs, the most widely used are the National Institutes of Health (NIH) consensus classification ^44^, Armed Forces Institute of Pathology (AFIP) criteria ^45^, and the “modified NIH” classification ^42^. These three classifications were recently found to have roughly similar prognostic accuracy in a series of 2,560 patients with resected GISTs who never received adjuvant imatinib ^39^. Notably, regardless of the classification scheme used, patients identified as intermediate risk had a clinical course similar to that of the low-risk group.

## Does adjuvant imatinib prolong recurrence-free survival or OS compared to placebo?

Three randomized phase III clinical trials (see Table 1) have examined the use of imatinib 400 mg daily as an adjuvant for 1, 2, and 3 years ^46– 48^; all three showed that it extends recurrence-free survival (RFS) in comparison with placebo or surveillance.

**Table 1.** Phase III clinical trials of adjuvant imatinib therapy.

Duration	3 years of adjuvant IM	2 years of adjuvant IM	1 year of adjuvant IM
Trial	SSGXVIII/AIO	EORTC 62024	ACOZOG Z9001
IM dosage	400 mg/day for 1 versus 3 years	400 mg/day for 2 years versus control (no IM)	400mg/day for 1 year versus control (no IM)
IM duration	MFU=54 months	MFU=4.7 years	MFU=19.7 months
Patients	n=400 Resected GIST (R0) with high risk of recurrence	N=908 KIT-positive completely resected GIST with intermediate or high risk of recurrence	KIT-positive after complete resection, T >3 cm, with low, intermediate, or high risk of recurrence
Primary end-point	RFS	Time to secondary resistance	RFS
Efficacy results	RFS at 5 years: 65.6% (3 years) versus 47.9% (1 year) ( *p*<0001) OS at 5 years: 92% (3 years) versus 81.7% (1 year) (p<0.02) 2d analysis	RFS at 3 years: 84% (2 years) versus 66% (control) OS at 5 years: 100% (2 years) versus 99% (control)	98% for IM versus 83% for placebo HR 0.35, *p*<0.0001 No significant difference in 1-year OS

GIST, gastrointestinal stromal tumor; HR, hazard ratio; IM, imatinib; MFU, median follow-up; OS, overall survival; RFS, recurrence-free survival

Additionally, the initial and long-term results provided by the AIO study ^47, 49^ demonstrated that 3 years of imatinib significantly improves RFS and OS compared with 1 year of therapy.

According to survival findings in the AIO trial, 3 years of adjuvant imatinib therapy are recommended for patients with GIST with high-risk features. To investigate whether the survival benefits have persisted, the authors performed the second planned analysis of the trial ^49^. In this second analysis ^49^, with a median FU of 90 months, patients randomized to 3-year imatinib dosing had longer disease control than those assigned to the 1-year dosing group; the relapse-free survival (RFS) rate after 5 years was 71% versus 52%, respectively (HR 0.60; 95% CI 0.44–0.81; *p*<0.001), and OS was also significantly improved: 92% versus 85% (HR 0.60; 95% CI 0.37–0.97; *p*=0.036) ^49^.

Despite the AIO trial survival findings, the optimal duration of adjuvant therapy remains unknown and is still being investigated. There is one non-randomized phase II trial of 5 years of adjuvant imatinib treatment that enrolled 91 high-risk GIST patients and showed that chronic imatinib was effective in preventing recurrences during treatment for patients with sensitive mutations; it also demonstrated 5-year RFS and OS rates of 90% and 95%, respectively, but 49% of patients discontinued treatment early ^50^. Moreover, two randomized trials are ongoing in high-risk GIST patients: a Scandinavian study comparing 5 years ^51^ with 3 years and a French study comparing 6 years ^52^ to 3 years of imatinib.

## Do all patients benefit from adjuvant imatinib?

A study that retrospectively analyzed Z9001 trial data proposed that high-risk patients derived greater efficacy from adjuvant therapy (tumor size >10 cm and high mitotic rate) ^46^. The use of adjuvant imatinib is not recommended for low risk and very low risk, but there is no consensus for intermediate risk ^53^. In this situation, the risks and benefits of treatment should be shared with the patient. A randomized phase II trial is ongoing in France (GIGIST study) comparing imatinib over 3 years versus surveillance in intermediate-risk patients with a high-risk genomic grade index ^54^.

Recent data from the Z9001 trial shed light on the efficacy of imatinib in different mutational subtypes ^55^. Imatinib was superior to placebo in prolonging RFS only in patients with deletions in *KIT* exon 11. It did not delay recurrence compared to placebo in *KIT* exon 11 point mutations or insertions, exon 9 mutations, or wild-type GIST ^55^. Imatinib was not statistically superior to placebo in *PDGFRA*-mutant tumors; however, the sample size was small. Similar data have been reported in the AIO trial examining the effect of 1 versus 3 years of imatinib ^47^. A meta-analysis of the three phase III randomized studies would be useful and instructive to have a better idea of the genotypes that benefit most or least from imatinib as adjuvant.

## Does adjuvant imatinib affect the development of imatinib resistance?

Results from the EORTC phase III trial ^48^ examining 2 years of adjuvant imatinib compared to surveillance, with a median FU of 4.7 years, reported that the time to initiation of a different TKI following recurrence (i.e. imatinib failure-free survival, IFFS), a surrogate for secondary resistance, was equivalent in both treatments arms (5-year IFFS was 87% in the imatinib arm versus 84% in the control arm; HR 0.79; *p*=0.21) ^41^. RFS was 84% versus 66% at 3 years and 69% versus 63% at 5 years (log rank *p*<0.01). The AIO trial also demonstrated no difference in time to progression following salvage imatinib in patients initially treated with either 1 or 3 years of adjuvant imatinib ^47^, suggesting that there is no evidence that the duration of prior adjuvant therapy has an impact on the TSR in the advanced setting.

## Imatinib in the neoadjuvant setting

Imatinib demonstrates high response rates in patients with metastatic GIST; therefore, the purpose of its preoperative use is in tumor bulk reduction in order to ease complete surgical resection or make organ preservation more likely in initially unresectable or borderline resectable disease. In the absence of phase III trials, the role of neoadjuvant imatinib therapy remains investigational, but data from retrospective series and a few prospective phase II trials have demonstrated the efficacy and safety of neoadjuvant imatinib in locally advanced GISTs ^56– 58^. Patient selection and duration of therapy are currently at the discretion of the medical oncologist and surgeon. NCCN and ESMO guidelines ^1, 2^ recommend neoadjuvant imatinib in patients who have primary, unresectable tumors or resectable tumors with a risk of significant morbidity but with an early tumor response assessment so that surgery is not delayed in case of non-responding disease. An initial dose of 400 mg daily is indicated; however, patients with exon 9 mutations may benefit from dose escalation. Imatinib should be continued for 6–9 months but not extended beyond 12 months because of the risk of imatinib resistance and of usually minor additional tumor shrinkage ^56, 59^.

## Recent advances: new promising drugs

In the last 10 years or so, research has yielded the discovery and greater understanding of the biological mechanisms behind GIST survival and proliferation. Only recently, a more comprehensive molecular analysis has shown that *KIT/PDGFRA* wild-type GIST is a rather heterogeneous group of different diseases rather than one single entity ^60^.

Between 20 and 40% of all *KIT/PDGFRA* wild-type GISTs are SDH-deficient, as recognized by the loss of SDH subunit B (SDHB) protein expression, which is most often due to germline and/or somatic loss-of-function mutations in any of the four SDH subunits (A, B, C, or D) ^61, 62^. These tumors are designated as SDH-deficient GISTs or SDHB-negative GISTs ^63^. The most common subtype of SDH-deficient GIST is made up of tumors with SDHA mutations ^63^.

The subgroup of the remaining *KIT/PDGFRA* wild-type, but not SDH-deficient, GISTs have been further characterized: about 15% possess an activating mutation in *BRAF* or, more infrequently, a *RAS* gene ^64^. Furthermore, wild-type GISTs may appear in the setting of syndromic neurofibromatosis type I (NF1) disease, in which there is a loss of function of the NF1 protein ^65^. Together, GISTs with mutations in *BRAF/RAS* or NF1 are known as RAS pathway (RAS-P) mutant GISTs. Approximately 5% of all GISTs lack functionally relevant mutations in *KIT PDGFR, BRAF*, or NF1 without deficient SDH complexes (as shown by maintaining expression of SDHB by immunohistochemistry); the oncogenic drivers of “quadruple negative” GIST remain uncertain and possibly more complex than other larger classes of GIST ^60, 63^. Some of these may be explained by newly identified oncogenic fusions in NTRK.

These last 15 years have led to the approval of three drugs (imatinib, sunitinib, and regorafenib) for the treatment of advanced GISTs, which have significantly improved survival. However, nearly all patients with metastatic GISTs will become resistant to those therapies. Various new targeted therapies are under evaluation in clinical trials for imatinib-resistant GISTs, with major interest in new promising multi-kinase inhibitors such as ponatinib, BLU-285, and crenolanib.

Ponatinib, a next-generation TKI approved in imatinib-resistant BCR-ABL leukemia, has shown activity in engineered and GIST-derived cell lines, potently inhibits *KIT* exon 11 primary mutants and a range of secondary mutants, including those within the A-loop, and has been shown to induce regression in engineered and GIST-derived tumor models containing these secondary mutations ^66^. The preliminary results from a non-randomized phase II trial that evaluated ponatinib at a dose of 45 mg/day in heavily treated GIST patients (74% had four or more prior agents) demonstrated a clinical benefit rate (CR, PR, or SD ³ 16 weeks) of 55% in patients with primary *KIT* exon 11 mutation, but responses were also observed with the 30 mg dose ^67^.

More recently, BLU-285, a new investigational agent, has shown highly potent and selective targeting of *KIT/PDGFRA* GIST mutants and high activity against imatinib-resistant GIST patient-derived xenografts of a *KIT* exon 11/17 mutant and a *KIT* exon 11/13 mutant ^68^. BLU-285 is a mutation-specific inhibitor of kinases with mutations in *KIT* D816V and *PDGFRA* D842V, in which most TKIs are ineffective. It appears to have favorable toxicity, as indicated by preclinical data, but we await the results of clinical trials ^69^. The results of the dose escalation part of a phase I study were recently presented at the 2017 ASCO meeting ^70^ and showed that BLU-285 is well tolerated on a QD schedule at doses up to the MTD of 400 mg and that its exposure at 300–400 mg QD provides broad coverage of primary and secondary *KIT/PDGFRA* mutants. BLU-285 has strong clinical activity in *PDGFRA* D842-mutant GISTs with an ORR of 60% per central review, and median PFS was not reached. It also demonstrates important anti-tumor activity including radiographic response and prolonged PFS in heavily pre-treated, *KIT*-mutant GISTs at doses of 300–400 mg QD ^70^. Based on these encouraging data, planning is underway for a phase III randomized study of BLU-285 in third-line metastatic GISTs.

The oral small-molecule inhibitor crenolanib exhibits activity against FLT3 and the PDGFRs (including D842V-mutated kinase) ^71^. Metastatic *PDGFRA*-mutant GIST is exceptionally unusual, and a phase II trial with seven patients demonstrated objective response in one and SD in three ^72^. A randomized, double-blind, placebo-controlled, multicenter, phase III trial of crenolanib in subjects with advanced or metastatic GIST with D842V mutation in the *PDGFRA* gene is ongoing ^73^.
